# Supplementary material for: Genome sequence of the model rice variety KitaakeX
Source: BMC Genomics. 2019 Nov 27;20:905. doi: 10.1186/s12864-019-6262-4 (PMC6882167; doi:10.1186/s12864-019-6262-4)
Supplement: Supplementary file 2 — Additional file 2: Table S7. BUSCO analysis of KitaakeX and comparison with other rice genomes. Table S8. Summary of transposable elements in KitaakeX, Nipponbare, and Zhenshan97. Table S9. Comparison of SNPs and INDELs between three rice genomes. Table S10. Comparison of single base substitutions between three rice genomes. Table S11. Oryza sativa KitaakeX annotation v3.1 on assembly v3.0. Table S12. Sequence length of pseudomolecules, number of genes and gene models for each of the 12 rice chromosomes. [file 12864_2019_6262_MOESM2_ESM.docx]

Additional file 2

**Table S7**: BUSCO analysis of KitaakeX and comparison with other rice genomes.

|  | KitaakeX | Nipponbare* | Zhenshan97* |
| --- | --- | --- | --- |
| Protein-coding genes | 35,594 | 39,045 | 34,610 |
| Complete BUSCOs (%) | 99.0 | 97.0 | 91.8 |
| Fragmented BUSCOs (%) | 0.3 | 1.0 | 0.8 |
| Missing BUSCOs (%) | 0.4 | 1.4 | 7.4 |

*Data are from Zhang et al (Zhang et al., 2016).

**Table S8:** Summary of transposable elements in KitaakeX, Nipponbare, and Zhenshan97

| TE classification | KitaakeX | | | | Nipponbare* | | | | Zhenshan97* | | | | |  |
| --- | --- | --- | --- | --- | --- | --- | --- | --- | --- | --- | --- | --- | --- | --- |
|  | Length (kb) | | Percentage (%) | | Length (kb) | | Percentage (%) | | | Length (kb) | | Percentage (%) | |  |
| **Retrotransposons** | | 89,620 | | 23.49 | | 84,546 | | 22.59 | | | 92,184 | | 25.61 | |
| Copia | 13,147 | | 3.45 | | 11,346 | | 3.03 | | | 9,462 | | 2.73 | |  |
| Gypsy | 70,797 | | 18.55 | | 66,689 | | 17.82 | | | 72,938 | | 21.03 | |  |
| LINEs | 3,992 | | 1.05 | | 3,350 | | 0.90 | | | 3,102 | | 0.86 | |  |
| SINEs | 374 | | 0.10 | | 1,426 | | 0.38 | | | 1,317 | | 0.37 | |  |
| Others | 1,310 | | 0.34 | | 1,735 | | 0.46 | | | 1,340 | | 0.37 | |  |
| **DNA transposons** | 32,552 | | 8.53 | | 63,146 | | 16.87 | | | 57,027 | | 15.84 | |  |
| Tc1-Mariner | 1,463 | | 0.38 | | 9,689 | | 2.59 | | | 8,940 | | 2.49 | |  |
| hAT | 1,792 | | 0.47 | | 4,337 | | 1.16 | | | 3,958 | | 1.10 | |  |
| Mutator | 7,484 | | 1.96 | | 15,736 | | 4.20 | | | 14,675 | | 4.08 | |  |
| PIF–Harbinger | 2,855 | | 0.75 | | 11,959 | | 3.19 | | | 11,126 | | 3.09 | |  |
| CACTA | 12,167 | | 3.19 | | 14,404 | | 3.85 | | | 11,925 | | 3.31 | |  |
| Helitron | 4,514 | | 1.18 | | 3,375 | | 0.90 | | | 3,081 | | 0.86 | |  |
| Others | 2,278 | | 0.59 | | 4,091 | | 1.09 | | | 11,695 | | 1.05 | |  |
| **Total** | 122,172 | | 32.02 | | 148,140 | | 39.58 | | | 149,662 | | 41.58 | |  |

*Data are from Zhang et al (Zhang et al., 2016).

**Table S9:** Comparison of SNPs and INDELs between three rice genomes

|  | KitaakeX vs. Nipponbare | KitaakeX vs. ZS97 | Nipponbare vs.  ZS97* |
| --- | --- | --- | --- |
| SNPs | 253,295 | 2,328,319 | 2,665,280 |
| SNPs/kb | 0.67 | 6.17 | 7.14 |
| INDELs | 75,183 | 442,962 | 486,015 |
| INDELs/kb | 0.20 | 1.17 | 1.31 |

*Data are from the ZS97 genome paper (Zhang et al., 2016).

**Table S10:** Comparison of single base substitutions between three rice genomes

| Substitution | KitaakeX vs. Nipponbare | KitaakeX vs. ZS97 | Nipponbare vs.  ZS97* |
| --- | --- | --- | --- |
| A->G | 43,744 | 390,891 | 447,240 |
| G->A | 42,497 | 436,480 | 497,244 |
| C->T | 42,375 | 434,854 | 495,844 |
| T->C | 44,114 | 392,605 | 449,693 |
| A->C | 10,004 | 87,253 | 99,660 |
| C->A | 10,566 | 91,047 | 103,398 |
| A->T | 11,150 | 93,603 | 110,671 |
| T->A | 11,597 | 95,297 | 112,538 |
| C->G | 8,049 | 63,856 | 72,939 |
| G->C | 8,538 | 64,714 | 73,645 |
| G->T | 10,092 | 90,079 | 102,695 |
| T->G | 10,569 | 87,640 | 99,713 |
| Transition | 172,730 | 1,654,830 | 1,890,021 |
| Transversion | 80,565 | 673,489 | 775,259 |
| Ti/Tv | 2.14 | 2.46 | 2.44 |

*Data are from the ZS97 genome paper (Zhang et al., 2016).

**Table S11:** Oryza sativa KitaakeX annotation v3.1 on assembly v3.0

| Primary transcripts (loci) | 35,594 |
| --- | --- |
| Alternative transcripts | 12,900 |
| Total transcripts | 48,494 |
|  |  |
| **For primary transcripts:** |  |
| Average number of exons | 4.7 |
| Median exon length | 178 |
| Median intron length | 153 |
|  |  |
| **Gene model support (value is number of gene models):** |  |
| Any EST support | 31,854 |
| EST support over 100% of their lengths | 29,039 |
| EST support over 95% of their lengths | 29,511 |
| EST support over 90% of their lengths | 29,819 |
| EST support over 75% of their lengths | 30,334 |
| EST support over 50% of their lengths | 30,861 |
| Pfam annotation | 23,583 |
| Panther annotation | 23,142 |
| KOG annotation | 12,696 |
| KEGG Orthology annotation | 8,290 |
| E.C. number annotation | 8,948 |

**Table S12:** Sequence length of pseudomolecules, number of genes and gene models for each of the 12 rice chromosomes

| Chromosome | Sequence length in pseudomolecule (bp) | Genes | Gene models |
| --- | --- | --- | --- |
| Chr1 | 44294598 | 4895 | 6750 |
| Chr2 | 37184994 | 3919 | 5357 |
| Chr3 | 37804764 | 4171 | 5745 |
| Chr4 | 36974593 | 3125 | 4290 |
| Chr5 | 30273398 | 2762 | 3809 |
| Chr6 | 31052574 | 2897 | 3941 |
| Chr7 | 29683244 | 2725 | 3676 |
| Chr8 | 29331338 | 2475 | 3369 |
| Chr9 | 22792385 | 1959 | 2631 |
| Chr10 | 24066873 | 2055 | 2717 |
| Chr11 | 29738898 | 2378 | 3255 |
| Chr12 | 27153574 | 2172 | 2888 |
| Total^a^ | 380351233 | 35533 | 48428 |

^a^In addition to the 12 chromosomes shown here, we found 61 loci and 66 gene models on other scaffolds. These additional loci and gene models are not included in the total shown here for the 12 main pseudomolecules.
