# Supplementary material for: Genome sequence of the model rice variety KitaakeX
Source: BMC Genomics. 2019 Nov 27;20:905. doi: 10.1186/s12864-019-6262-4 (PMC6882167; doi:10.1186/s12864-019-6262-4)
Supplement: Supplementary file 7 — Additional file 7: Figure S16. Genomic variation showing gene variations between KitaakeX and Nipponbare and ZS97. a. Length distribution of InDels in protein-coding regions. b. SNPs and InDels that cause high-impact gene variations between KitaakeX and Nipponbare and ZS97. c. Gene enrichment in KitaakeX unique present regions compared with Nipponbare. [file 12864_2019_6262_MOESM7_ESM.docx]

**
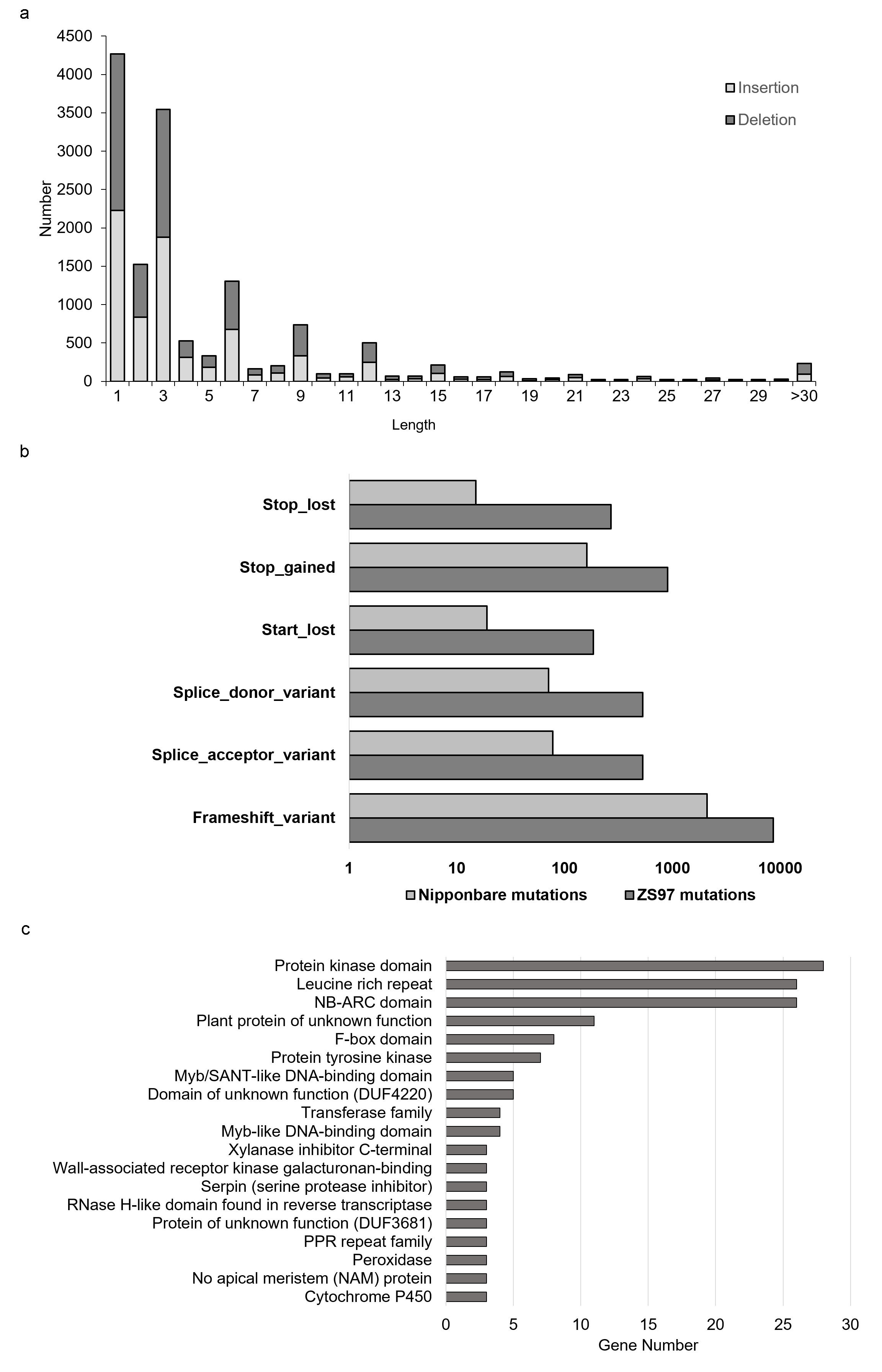
Figure S16:** Genomic variation showing gene variation between KitaakeX and Nipponbare and ZS97. ***a*** Length distribution of InDels in protein-coding regions. The distribution indicates that nonsingle base InDels with a length of 3 bp (and/or multiples of 3) are much more abundant than the others. ***b*** SNPs and InDels that cause high-impact gene variations between KitaakeX and Nipponbare and ZS97. ***c*** Gene enrichment in KitaakeX unique present regions compared with Nipponbare.

pentatricopeptide repeat (PPR), NB-ARC (Nucleotide Binding)- (APAF-1 (apoptotic protease-activating factor-1), R proteins and CED-4 (*Caenorhabditis elegans* death-4 protein)),SANT domain (Swi3, Ada2, N-Cor, and TFIIIB).
