# Supplementary material for: Genome sequence of the model rice variety KitaakeX
Source: BMC Genomics. 2019 Nov 27;20:905. doi: 10.1186/s12864-019-6262-4 (PMC6882167; doi:10.1186/s12864-019-6262-4)
Supplement: Supplementary file 9 — Additional file 9: Figure S17. Integrative genomics viewer (IGV) snapshot showing presence of XA21 transgene and selectable marker encoding a hygromycin B phosphotransferase on chromosome 6 of KitaakeX. [file 12864_2019_6262_MOESM9_ESM.docx]

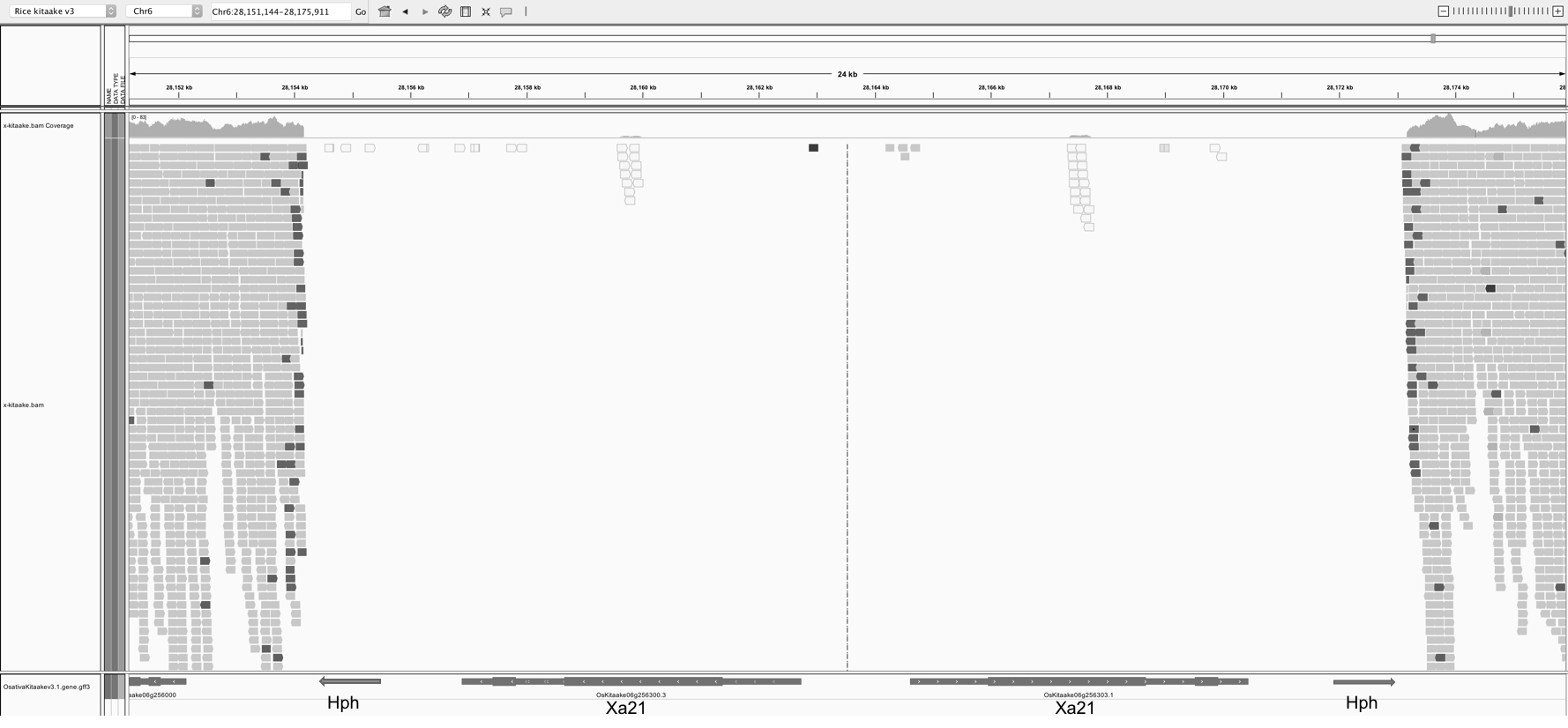


**Figure S17:** Integrative genomics viewer (IGV) snapshot showing presence of XA21 transgene and selectable marker encoding a hygromycin B phosphotransferase on chromosome 6 of KitaakeX.
